# Unraveling causal pathways in retinal vein occlusion: a systematic review of Mendelian randomization studies

**DOI:** 10.1186/s40942-025-00753-7

**Published:** 2025-12-03

**Authors:** Anas Alamoudi, Ahmed Alnabihi, Sultan Al-Qahtani, Abdulaziz Aiyidh S. Aljiayyd, Waleed K. Alsarhani, Suzan Y. Alharbi, Andrew Mihalache, Marko Popovic, Rajeev H. Muni, Adel G. Alakeely

**Affiliations:** 1 Department of Ophthalmology, Jeddah Eye Hospital, Jeddah, Saudi Arabia; 2 College of Medicine, King Saud bin Abdulaziz University for Health Sciences, Jeddah, Saudi Arabia; 3 Department of Ophthalmology, King Khaled Eye Specialist Hospital and Research Center, Riyadh, Saudi Arabia; 4 Department of Ophthalmology and Vision Sciences, University of Toronto, Toronto, Ontario Canada; 5 Department of Ophthalmology, King Faisal Specialist Hospital & Research Centre, Riyadh, Saudi Arabia; 6 Temerty Faculty of Medicine, University of Toronto, Toronto, Ontario Canada; 7 Department of Ophthalmology, St. Michael’s Hospital/Unity Health Toronto, Toronto, Ontario Canada; 8 Magrabi Hospitals and Centers, Riyadh, Saudi Arabia

**Keywords:** Retinal vein occlusion, Mendelian randomization, Risk, Hypertension, Glycemic traits

## Abstract

**Background:**

Retinal vein occlusion (RVO) is a leading cause of vision loss, yet there are inconsistent risk estimates related to risk factors. Mendelian randomization (MR) uses genetic variants as proxies for lifelong exposure and can clarify causal pathways for RVO. We aimed to systematically review MR studies to identify causally supported systemic and ocular risk factors for RVO.

**Methods:**

Four databases (PubMed, Embase, Scopus, and Web of Science) were searched from inception to June 2025 for peer-reviewed MR studies evaluating modifiable systemic or ocular risk factors in relation to any form of RVO utilizing GWAS data. Narrative synthesis was undertaken as methodological heterogeneity precluded meta-analysis. All effect estimates (ORs) were extracted directly from individual studies and robustness of evidence for each exposure across studies was assessed as robust, probable, suggestive, insufficient, and non-evaluable based on significance and direction of evidence.

**Results:**

Twelve two-sample MR studies, all conducted in European cohorts, met inclusion criteria. Ocular traits showed the most consistent signals: higher intraocular pressure (RVO (OR = 1.53, 95% CI: 1.0402.26) and glaucoma liability (OR = 1.31, 95% CI: 1.18–1.45) were robustly associated with greater risk of RVO. Among cardiovascular factors, elevated blood pressure/hypertension liability demonstrated probable evidence of increased RVO risk (OR = 1.58, 95% CI: 1.34–1.85), whereas lipid profiles yielded mixed signals, with some support for higher LDL (OR = 1.23, 95% CI: 1.05–1.44) and total cholesterol (OR = 1.44, 95% CI: 1.08–1.92) effects. For metabolic factors, glycemic traits showed probable to robust evidence with fasting glucose (OR = 5.01, 95% CI: 2.00-12.55) and two-hour glucose (OR = 3.17, 95% CI: 1.63–6.18) associated with higher RVO risk. Similarly, type 2 diabetes liability showed probable evidence (OR = 2.82, 95% CI: 2.07–3.85); anthropometric measures offered probable to robust support with body mass index (OR = 1.94, 95% CI: 1.23–3.08) and waist circumference (OR = 2.40, 95% CI: 1.36–4.24) associated with RVO. In other domains, selected coagulation and platelet traits showed probable-robust signals, vitamin D evidence was insufficient, and gut microbiota instruments provided preliminary robust evidence for Bacilli and Family XIII AD3011 association with RVO.

**Conclusion:**

Genetic evidence supports a multifactorial vascular-metabolic model for RVO in which elevated IOP, glaucoma, hypertension, adiposity, and acute hyperglycemia are genetically supported risk factors. These findings highlight blood-pressure control, weight management, and glycemic regulation as important prevention targets and underscore the need for ancestry-diverse MR studies with refined phenotyping.

**Supplementary Information:**

The online version contains supplementary material available at 10.1186/s40942-025-00753-7.

## Introduction

Retinal vein occlusion (RVO) is the second most common sight-threatening retinal vascular disorder after diabetic retinopathy, affecting an estimated 25 million people worldwide and occurring in roughly 15 of every 100,000 individuals each year [1, 2]. Central (CRVO) and branch (BRVO) variants differ in anatomic location but share an acute, often painless loss of vision and a propensity for sight-limiting sequelae such as macular edema, retinal and disc neovascularization, vitreous hemorrhage, and neovascular glaucoma [3]. Alongside the unavoidable contribution of aging, a wide array of systemic and ocular conditions including hypertension, type 2 diabetes mellitus (T2DM), dyslipidemia, glaucoma, obesity, coagulation abnormalities, and disturbed glycemic control- have been implicated in disease onset [4–6]. Yet for many of these factors, the literature remains inconsistent, with observational studies often reporting conflicting associations between these factors and disease onset [4]. These discrepancies largely arise because retrospective and cross-sectional designs cannot fully eliminate selection bias, confounding, reverse causation, or measurement error in modifiable exposures that fluctuate with medication use or lifestyle [7, 8].

Mendelian randomization (MR) offers a powerful way to disentangle correlation from causality by using germline genetic variants as unconfounded proxies for lifelong differences in an exposure. Since alleles are randomly assorted at conception, MR analyses are far less susceptible to the biases that undermine traditional epidemiology and can provide quasi-experimental evidence on etiological pathways [8, 9]. Over the past decade, large genome-wide association studies (GWAS) have yielded robust instrumental variables for blood pressure, lipid fractions, glycemic traits, adiposity indices, coagulation factors, vitamin D, and other potential drivers of vascular disease that has been used in identifying risk factors for ocular and non-ocular diseases [10, 11]. Isolated MR investigations have begun to investigate individual exposures in relation to RVO risk, but their findings remain scattered, occasionally contradictory, and have not been systematically synthesized to date.

The current systematic review therefore aims to synthesize and critically appraise all MR studies that evaluate any risk factor for RVO. By summarizing effect estimates across lipid traits, metabolic parameters, coagulation profiles, anthropometric measures, micronutrients, and other candidate exposures, we seek to clarify which associations are likely to be truly causal, highlight areas of consistent null evidence, and identify knowledge gaps that warrant further genetic or mechanistic investigation.

## Methods

This systematic review was conducted in line with the Cochrane Handbook for Systematic Reviews of Interventions and adhered to the PRISMA reporting standards (PROSPERO registration number: CRD420251077217) [12, 13].

### Eligibility criteria

We included human MR studies that explored potential causal associations between any exposure and RVO, including its subtypes: CRVO and BRVO. Eligible study designs were peer-reviewed MR analyses utilizing GWAS data. To be included, studies had to report the outcome as RVO or a recognized subtype and apply genetic instruments meeting standard MR assumptions. Non-MR designs, conference abstracts, reviews, commentaries, or in vitro/animal studies were excluded.

### Information sources and search strategy

A comprehensive literature search was performed across PubMed, Embase, Scopus, and Web of Science from database inception to June 4, 2025. We used a combination of controlled vocabulary and keyword terms targeting (i) MR methodologies and (ii) the occurrence of RVO. A summary of our search strategy for PubMed, Embase, Scopus, and Web of Science is presented in Supplemental Table 1. Equivalent strategies tailored to each database syntax were used. No restrictions were applied regarding language or publication year. Reference lists of all included studies and relevant reviews were manually screened to identify additional eligible studies.

### Study selection and data extraction

All retrieved records were first imported into EndNote 20 for de-duplication and then uploaded to Rayyan for independent screening [14]. Two reviewers (Alnabihi A and Aljiayyd AAS) conducted title/abstract screening followed by full-text review. Any disagreements were resolved through discussion or consultation with a third reviewer (Alamoudi A). Using a standardized Microsoft Excel extraction form, two reviewers independently collected data on study authors, publication year, evaluated exposures, MR design (e.g., one-sample or two-sample), GWAS datasets used, number and strength of genetic instruments, outcome definitions, effect estimates (odds ratios and confidence intervals), and sensitivity analyses. We also extracted methodological details including the primary MR estimator used (e.g., inverse variance weighted), sensitivity analyses performed (MR-Egger, weighted median/mode, MR-PRESSO, multivariable MR), SNP-inclusion thresholds (commonly *p* < 5 × 10⁻⁸, occasionally *p* < 5 × 10⁻⁶), and instrument strength (F-statistics).When necessary, study authors were contacted via email for missing or unclear information (two attempts spaced four weeks apart).

### Risk of bias (RoB) and quality assessment

Our appraisal centered on the three core MR assumptions: (1) relevance: genetic instruments must predict the exposure; (2) independence: instruments are uncorrelated with confounders; and (3) exclusion-restriction: their effect on the outcome operates solely through the exposure [9]. Violating the latter two (collectively, horizontal pleiotropy) is difficult to rule out, so we documented how each study addressed them and what sensitivity tests were applied. Study quality was judged using the STROBE-MR checklist [15]. Scores were converted to percentages and grouped as low (< 75%), moderate (75–85%), or high (> 85%) risk of bias. Because no universally accepted meta-analysis tool exists for MR bias, this checklist served as our primary standard.

There is no universally accepted tool to assess the grade of evidence of MR associations, so we opted to use tools developed by previous researchers. We pre-specified four evidence grades based on the concordance and statistical significance of the main MR result, plus ≥ 1 sensitivity method (MR-Egger, weighted median, MR-PRESSO, or multivariable MR) [16]:

### Grade: criteria (all p-values nominal unless study applied multiplicity correction)

Robust: All available methods are significant with identical effect direction.

Probable: ≥1 method significant and all directions consistent.

Suggestive: ≥1 method is significant, but effect directions conflict.

Insufficient: No method is significant.

Analyses lacking any sensitivity testing were labelled non-evaluable. Given the limited power of MR-Egger, we repeated the grading after omitting it; associations assessed solely by MR-Egger were then left ungraded [17]. This approach allowed us to better assess the overall state of evidence and the robustness of reported associations.

## Results

### Characteristics of included studies

**Fig. 1:**
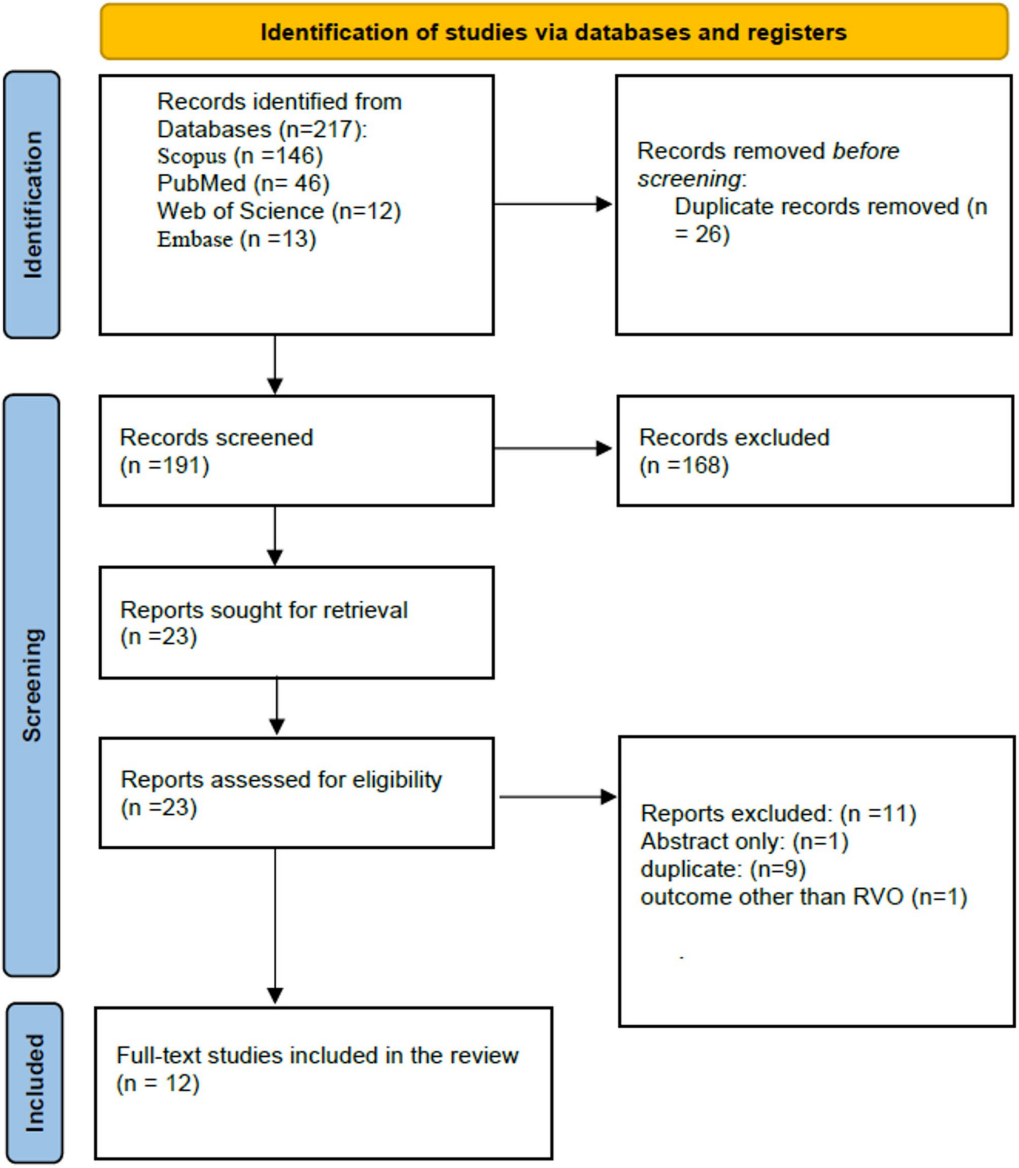
PRISMA flow diagram of the study selection process

Of the 217 records initially identified, 191 records were screened, and 12 studies were ultimately included in our systematic review (Fig. 1). The 12 MR studies we included were all two-sample MR analyses conducted in European-ancestry populations, with sample sizes for exposure GWAS (risk factor datasets) ranging from 10,701 to over 900,000 and outcome GWAS (RVO case datasets) from 372 to 1,595 (Table 1). Four studies investigated lipid and metabolic traits: high-density lipoprotein cholesterol (HDL-C), low-density lipoprotein cholesterol (LDL-C), triglycerides (TG), and total cholesterol (TC) [18], T2DM liability [19], serum 25-hydroxyvitamin D [25(OH)D] [20], and multi-lipid with blood-pressure traits [21]. Ophthalmic phenotypes were examined in three studies, assessing intraocular pressure (IOP) and primary open-angle glaucoma (POAG) [22], glaucoma [23]), and associations between hypertension and glaucoma [24]. One study explored bidirectional relationships between RVO and ischemic stroke/myocardial infarction (IS/MI) [10]. Additionally, single-study analyses assessed gut microbiota composition [25], glycemic traits [26], coagulation factors with platelets [27], as well as body mass index (BMI) and body-composition metrics [28]. All employed inverse variance weighted (IVW) as the primary estimator with extensive sensitivity checks (MR-Egger, weighted median/mode, MR-PRESSO, and, where applicable, MVMR), used stringent SNP-inclusion thresholds (generally *p* < 5 × 10⁸ or, in some studies, *p* < 5 × 10⁶), and reported strong instrument strength (all F-statistics > 10). RVO was uniformly defined via ICD-coded registry data (FinnGen), ensuring consistency in outcome ascertainment across studies. Across the 12 MR studies, all studies showed high reporting quality, between 85% and 93%, indicating broad adherence to STROBE-MR fundamentals. For further information refer to Table 2.

**Table 1:** Characteristics of included studies

Study ID	MR design	Datasets used	total sample size	exposure(s) evaluated	genetic instruments SNPs	instrument strength	outcome definition	effect estimates
Zheng 2022 [18]	Two-sample MR using IVW (primary), MR-Egger, weighted median; Multivariable MR adjusting for diabetes and lipids	Exposure: GLGC and UKB; Outcome: FinnGen ; European ancestry	GLGC: 188,577; UKB: 115,078; FinnGen: 204,703	HDL-C, LDL-C, total cholesterol, triglycerides (units not specified)	GLGC: HDL-C (86), LDL-C (77), TG (53), TC (81); UKB: HDL-C (78), LDL-C (45), TG (65), TC (57)	All F > 10; R² ranged 4.31%–9.41%	RVO, defined via registry data (FinnGen)	HDL-C: OR = 0.806 (0.659–0.986); LDL-C: OR = 1.233 (1.054–1.442); TC: MR-Egger OR = 1.441 (1.083–1.916); TG: OR = 1.103 (0.883–1.378)
Huang 2023 [19]	Two-sample MR; IVW (main), MR-Egger, weighted median, weighted mode, maximum likelihood, MR-PRESSO; multivariable MR	T2DM: Mahajan 2018 (Discovery), Bonàs-Guarch 2018 (Validation); Outcome: FinnGen; European	T2DM (Discovery): 48,286 cases / 250,671 controls; Validation: 12,931 cases / 57,196 controls; RVO: 372 cases / 182,573 controls	T2DM	Discovery: 67 SNPs; Validation: 16 SNPs; MVMR: 30 SNPs / 5 SNPs	F = 559.83 (Discovery), 685.05 (Validation); R² = 11.15% (Discovery)	ICD-coded diagnosis (FinnGen)	IVW: OR = 2.823 (95% CI: 2.072–3.847); Weighted Median: OR = 2.415 (1.411–4.132); Weighted Mode: OR = 2.370 (1.321–4.252); Maximum Likelihood: OR = 2.871 (2.100–3.924); MR-PRESSO: OR = 2.823 (2.135–3.733); MR-Egger: OR = 2.441 (1.149–5.184); MVMR (Discovery): OR = 1.748 (1.238–2.467); MVMR (Validation): OR = 1.433 (1.132–1.814)
Fan 2024 [20]	Two-sample MR; main method: IVW; sensitivity methods: MR-Egger, Weighted Median, MR-PRESSO, MVMR	Vitamin D: UK Biobank (Wang et al. 2023, Revez et al. 2020); RVO: FinnGen; European ancestry	Vitamin D (324,105 and 417,580); RVO (775 cases, 308,633 controls)	25(OH)D; units not reported	60–66 SNPs (after outlier exclusion), 227–238 SNPs in MVMR	All SNPs F > 10	RVO diagnosis based on FinnGen registry data (ICD-coded)	Discovery OR = 1.34 (95% CI: 0.80–2.23, *p* = 0.263); Validation OR = 1.27 (95% CI: 0.75–2.14, *p* = 0.375)
Katsimpris 2024 [22]	Two-sample MR; main analysis: IVW; sensitivity: MR-Egger, weighted median, MR-PRESSO; MR mediation to quantify POAG mediation	IOP: UK Biobank corneal-compensated GWAS POAG: GWAS meta-analysis; RVO: FinnGen R9	IOP GWAS: 97,653 POAG GWAS: 216,257 RVO GWAS: 377,277 (775 cases, 376,502 controls)	IOP (mmHg, corneal-compensated)	47 SNPs for IOP; 50 SNPs for POAG	IOP SNPs: F ≥ 29.96; R² = 2.64%	RVO diagnosed via ICD-9/ICD-10 codes in FinnGen registry	Total effect of IOP on RVO: OR = 1.53 (95% CI: 1.04–2.26; *p* = 0.03) Proportion mediated via POAG: 91.6%
Ortín Vela 2024 [21]	Two-sample MR; primary method: IVW; sensitivity: MR-Egger, weighted median, MR-PRESSO, multivariable MR for mediation	Exposures: ICBP (blood pressure traits), GLGC (lipids), Mahajan et al. (type 2 diabetes); Outcome: FinnGen R8 (retinal vein occlusion); All European ancestry	Exposure GWAS: ICBP (757,601), GLGC (188,577), Mahajan (898,130); Outcome GWAS: FinnGen R8 (~ 377,277 total, 6,127 RVO cases)	SBP (mmHg), DBP (mmHg), T2D (log odds), HDL-C, LDL-C, TG (mmol/L)	SBP: 302 SNPs; DBP: 301; HDL-C: 220; LDL-C: 197; TG: 215; T2D: 243	F-statistics > 10 across exposures (exact values not stated)	RVO defined using ICD codes (ICD-10: H34.8, H34.81, H34.83, H34.89) from FinnGen biobank-linked registry data	SBP→RVO: OR = 1.32 [1.16–1.49]; DBP→RVO: OR = 1.40 [1.18–1.66]; HDL-C→RVO: OR = 0.78 [0.66–0.91]; LDL-C→RVO: OR = 1.02 [0.85–1.22]; TG→RVO: OR = 1.18 [1.00–1.39]; T2D→RVO: OR = 1.17 [1.02–1.34]
Wang 2024 [23]	Two-sample MR; IVW (primary), MR-Egger, weighted median, weighted mode	Glaucoma: Craig et al.; European ancestry; RVO: FinnGen biobank, European ancestry	Glaucoma: 133,492 cases, 90,939 controls; RVO: 1,595 cases, 203,108 controls	Glaucoma (binary diagnosis via ICD codes and self-report)	85 SNPs	F > 30 for each SNP, R² = 1.4%	RVO diagnosed via registry and ICD codes (FinnGen)	IVW: OR = 1.306 (1.181–1.445); MR-Egger: OR = 1.476 (1.128–1.933); Weighted Median: OR = 1.235 (1.067–1.429); Weighted Mode: OR = 1.633 (1.261–2.116)
Zhang 2024 [29]	Bidirectional two-sample MR; IVW primary method; sensitivity analyses: MR-Egger, weighted median, MR-PRESSO, MR-RAPS, maximum likelihood	Exposure: FinnGen_R7 (European descent); Outcome: GIGASTROKE, CARDIoGRAMplusC4D, HERMES, UK Biobank, Neale lab (European descent)	RVO: 572 / 255,766; CRAO: 390 / 284,826; BRAO: 156 / 255,766	RVO, CRAO, BRAO (units not specified, genetically predicted liability)	*p* < 1e-5 for exposures; exact SNP number not in main text	F-statistics > 10; R² = 0.05%–1.06%	Diagnosed by ophthalmologists per international criteria; outcomes from clinical GWASs	RVO → IS: OR = 1.021 (95% CI: 1.004–1.037); CRAO → MI: OR = 1.014 (95% CI: 1.006–1.023); BRAO → Stroke: OR = 1.008 (95% CI: 1.002–1.013), IS: OR = 1.007 (95% CI: 1.001–1.014), CES: OR = 1.018 (95% CI: 1.006–1.031)
Jiang 2025 [24]	Two-sample MR; IVW (main), MR-Egger, weighted median; MVMR for hypertension and glaucoma	IEU Open GWAS Project; FinnGen for outcome; European ancestry	Exposure GWAS: 146,806–1,030,836; RVO: 1595 cases, 203,108 controls	Hypertension (logOR), Glaucoma (logOR), T2DM (logOR), Fasting Glucose (SD), Fasting Insulin (SD), HbA1c (SD), Stroke (logOR), MI (logOR), AF (logOR), WHR (SD), BMI (SD)	17–141 SNPs depending on exposure	F-statistics ranged from 40.46 to 139.78	ICD-based RVO in FinnGen	Hypertension: OR = 1.577 (1.342, 1.854); Glaucoma: OR = 1.24 (1.115, 1.379); T2DM: OR = 1.162 (1.045, 1.291); Others: NS
Lei 2025 [25]	Two-sample MR using IVW as primary method; weighted median and weighted mode as sensitivity analyses; pleiotropy and heterogeneity assessed by Cochran’s Q, MR-Egger, and MR-PRESSO	MiBioGen for gut microbiota (European ancestry); FinnGen and UK Biobank for RVO (European ancestry)	FinnGen: 309,408; UKB: 387,189	Class Bacilli, Order Lactobacillales, Family Streptococcaceae, Genus Clostridium innocuum group, Genus Family XIII AD3011 group, Genus Subdoligranulum (units not reported)	NR	F-statistic > 10	ICD-10 code H34.8 (includes CRVO and BRVO)	Bacilli: OR 1.34 (1.12–1.59); Lactobacillales: OR 1.31 (1.09–1.59); Streptococcaceae: OR 1.46 (1.08–1.99); Clostridium innocuum: OR 1.17 (1.02–1.34); Family XIII AD3011: OR 5.10 (2.05–12.65); Subdoligranulum: OR 1.31 (1.06–1.61)
Nie 2025 [26]	Two-sample MR; methods used: IVW (main), MR-Egger, weighted median, simple model, weighted model; pleiotropy tested with MR-Egger and MR-PRESSO; heterogeneity with Cochran’s Q	Exposures: IEU OpenGWAS – adiponectin, BMI, fasting glucose, fasting insulin, HbA1c, proinsulin (*n* = 10,701); Outcomes: IEU OpenGWAS – inflammatory eye diseases including RVO; ancestry: mixed European	Adiponectin: 39,883; BMI: 315,347; Fasting Glucose: 58,074; Fasting Insulin: 108,557; HbA1c: 46,368; Proinsulin: 10,701	Adiponectin, BMI, Fasting glucose, Fasting insulin, HbA1c, Proinsulin	NR	Steiger test passed; no F-stat or R² reported	Retinal vein occlusion (central and branch); ascertained via GWAS summary data	HbA1c → RVO: OR = 0.266, 95% CI = 0.105–0.675
Yuan-a 2025 [27]	Two-sample MR; IVW (primary), Weighted Median, MR-Egger	IEU Open GWAS (European ancestry); Outcome from FinnGen (European ancestry)	Exposure GWAS varies; Outcome GWAS: 372 RVO cases, 182,573 controls	FIII, FV, FVII, FVIII, FXI, FX, plasmin, platelet count, PCT, MPV, PDW (no unit transformation)	FIII (5), FVII (3), FVIII (11), MPV (9), PCT (6), FV (4), FX (3), FXI (7), plasmin (4), platelet count (8), and PDW (6).	F-statistics > 10	RVO diagnosis from FinnGen registry data	FIII: OR 0.577 (0.346–0.962), FVII: OR 0.742 (0.568–0.969), FVIII: OR 1.308 (1.067–1.604), MPV: OR 0.783 (0.621–0.987), PCT: OR 0.740 (0.560–0.977)
Yuan-b 2025 [28]	Two-sample MR; primary: IVW; sensitivity: MR-Egger, weighted median, MVMR	Exposures: IEU Open GWAS (European ancestry); Outcome: FinnGen (European ancestry)	Exposures vary (e.g., BMI: 681,275); Outcome: RVO: 372 cases, 182,573 controls	BMI, WC, HC, WHR, body fat %, whole-body fat-free mass, fasting glucose, 2 h-glucose, fasting insulin, HbA1c (all per 1 SD)	BMI: 423, Waist circumference: 247, Body fat %: 135, Fat-free mass: 191, Fasting glucose: 27, Two-hour glucose: 11	All F-statistics > 10 (e.g., BMI: 29–1426); R² per exposure	ICD-10 code H34 in FinnGen database	BMI (1.94 [1.23–3.08]); WC (2.4 [1.36–4.24]); FG (5.01 [2–12.55]); 2 h-glucose (3.17 [1.63–6.18]); FFM (0.45 [0.26–0.8])

MR = Mendelian Randomization; IVW = Inverse Variance Weighted; MR-Egger = Mendelian Randomization Egger regression; WM = Weighted Median; MVMR = Multivariable Mendelian Randomization; MR-PRESSO = Mendelian Randomization Pleiotropy RESidual Sum and Outlier; MR-RAPS = Robust Adjusted Profile Score; GWAS = Genome-Wide Association Study; UKB = UK Biobank; GLGC = Global Lipids Genetics Consortium; ICBP = International Consortium for Blood Pressure; T2DM = Type 2 Diabetes Mellitus; HDL-C = High-Density Lipoprotein Cholesterol; LDL-C = Low-Density Lipoprotein Cholesterol; TG = Triglycerides; TC = Total Cholesterol; IOP = Intraocular Pressure; POAG = Primary Open-Angle Glaucoma; RVO = Retinal Vein Occlusion; CRAO = Central Retinal Artery Occlusion; BRAO = Branch Retinal Artery Occlusion; IS = Ischemic Stroke; MI = Myocardial Infarction; AF = Atrial Fibrillation; WHR = Waist-Hip Ratio; BMI = Body Mass Index; WC = Waist Circumference; HC = Hip Circumference; FFM = Fat-Free Mass; SD = Standard Deviation; SNP = Single Nucleotide Polymorphism; OR = Odds Ratio; CI = Confidence Interval; ICD = International Classification of Diseases; NR = Not reported

**Table 2:** Risk of bias (RoB) and reporting quality of included MR studies using the STROBE-MR checklist

Author	Title and Abstract	Background	Objectives	Study Design and Data Sources	Assumptions	Statistical Methods: Main Analysis	Assessment of Assumptions	Sensitivity and Additional Analysis	Software and Pre-Registration	Descriptive Data	Main Results	Assessment of Assumptions (Results)	Sensitivity and Additional Analysis (Results)	Key Results	Limitations	Interpretation	Generalizability	Funding	Data and Data Sharing	Conflicts of Interest	Total Score (out of 20)	Score (%)
Lei 2025	1	1	1	1	0.5	1	1	1	0.5	1	1	1	1	1	1	1	0.5	1	0.5	1	18	90
Wang 2024	1	1	1	1	1	1	1	1	0.5	1	1	1	1	1	1	1	0.5	1	0.5	1	18.5	92.5
Jiang 2025	1	1	1	1	0.5	1	1	1	0.5	1	1	1	1	1	1	1	0.5	1	0.5	1	17.5	87.5
Katsimpris 2024	1	1	1	1	1	1	1	1	0.5	1	1	1	1	1	1	1	0.5	1	0.5	1	18	90
OrtÃn-Vela 2024	0	1	1	1	0.5	1	1	1	0.5	1	1	1	1	1	1	1	0.5	1	0.5	1	17	85
Nie 2025	1	1	1	1	0.5	1	1	1	0.5	1	1	1	1	1	1	1	0.5	1	0.5	1	17.5	87.5
Yuan-a 2025	1	1	1	1	1	1	1	1	0.5	1	1	1	1	1	1	1	0.5	1	0.5	1	18.5	92.5
Yuan-b 2025	1	1	1	1	1	1	1	1	0.5	1	1	1	1	1	1	1	0.5	1	0.5	1	18.5	92.5
Zheng 2022	1	1	1	1	1	1	1	1	0.5	1	1	1	1	1	1	1	0.5	1	0.5	1	18.5	92.5
Huang 2023	1	1	1	1	1	1	1	1	0.5	1	1	1	1	1	1	1	0.5	1	0.5	1	18.5	92.5
Fan 2024	1	1	1	1	1	1	1	1	0.5	1	1	1	1	1	1	1	0.5	1	0.5	1	18.5	92.5
Zhang 2024	1	1	1	1	0.5	1	1	1	0.5	1	1	1	1	1	1	1	0.5	1	0.5	1	17.5	87.5

Footnotes: 1 = fully reported, 0.5 = partially reported, 0 = not reported. Scores were calculated using the STROBE-MR checklist (max = 20 points). Quality categories: High (> 85%), Moderate (75–85%), Low (< 75%)

### Ocular risk factors

Several MR studies evaluated vascular, metabolic, and ocular risk factors for RVO. Katsimpris et al. (2024) employed 47 strong, genome-wide-significant SNPs for corneal-compensated IOP and 50 SNP instruments for POAG and showed that each genetically predicted 1 mmHg rise in IOP increased RVO risk by 53% (OR = 1.53, 95% CI 1.04–2.26), with MR-Egger, weighted-median, MR-PRESSO, and leave-one-out sensitivity checks yielding concordant results and no evidence of pleiotropy or weak instruments [22]. For clarification, these estimates were derived from genetic instruments for IOP and glaucoma liability. The RVO outcome datasets were not restricted to patients with clinically diagnosed glaucoma. Therefore, the proportion of RVO patients with glaucoma cannot be determined from these analyses. A two-step multivariable MR indicated that 91.6% of the IOP effect on RVO is mediated through POAG, suggesting that elevated IOP chiefly heightens RVO risk via glaucomatous optic-nerve changes rather than a direct hemodynamic pathway. This was further supported by the study Wang et al., which used 85 genome-wide-significant SNPs as instruments for a binary exposure of glaucoma and found that genetically predicted glaucoma was associated with a 30% higher odds of RVO (OR = 1.31, 95% CI 1.18–1.45) [23]. Sensitivity methods were directionally consistent—weighted-median OR = 1.24 (95% CI1.07-1.43), weighted-mode OR = 1.63 (95% CI 1.26–2.12), while MR-Egger produced a slightly wider but still positive estimate (OR = 1.48, 95% CI 1.13–1.93). Instrument strength was robust (F > 30; R² ≈ 1.4%), heterogeneity was low, and no evidence of horizontal pleiotropy was detected, supporting a moderate, causal elevation in RVO risk attributable to lifelong genetically elevated glaucoma liability.

### Cardiovascular and blood pressure traits

Jiang et al. conducted an MR analysis and found significant causal associations of genetically predicted hypertension and glaucoma with increased RVO risk [24]. Specifically, a one standard deviation (SD) increase in genetically predicted hypertension was associated with a 58% higher odds of RVO (OR = 1.58, 95% CI: 1.34–1.85), which was further supported by multivariable MR adjusting for glaucoma (OR = 1.78, 95% CI: 1.49–2.13). Similarly, genetically predicted glaucoma was associated with a 24% increased odds of RVO (OR = 1.24, 95% CI: 1.12–1.38), remaining significant after adjustment for hypertension (OR = 1.30, 95% CI: 1.18–1.44). Weak evidence suggested a potential association of T2DM with RVO risk (OR = 1.16, 95% CI: 1.05–1.29). Other evaluated exposures, including obesity measures, glycemic traits, and cardiovascular events, showed no causal effect.

In addition, Zhang et al. found reported modest but statistically significant positive associations between genetic liability to RVO and ischemic stroke (OR = 1.02, 95% CI: 1.00–1.04) [29]. Notably, no reverse causal effect of cardiovascular diseases (CVDs) on retinal vascular occlusions was observed.

The findings of Orten Vela et al. further suggest that people with genetically higher systolic and diastolic blood pressure had strong, consistent effects, with each 10-mmHg increase linked to approximately 0.15 SD reductions in arterial vascular density and increases of about 0.12 SD in retinal vessel tortuosity [21]. Higher BMI also showed a notable impact, with a 1-SD increase in BMI causing roughly a 0.18-SD rise in venous diameter variability. Lipid exposures such as HDL-C and LDL-C showed smaller and less consistent associations.

### Glycemic and obesity traits

Findings for metabolic traits varied across studies. Jiang et al. did not identify a significant relationship between glycemic traits and RVO [24]. In contrast, Nie et al. reported that lower genetically predicted HbA1c was associated with a statistically significant protective effect on RVO risk, showing a markedly reduced risk (OR = 0.25; 95% CI: 0.11–0.68). Other glycemic traits did not demonstrate significant causal associations with RVO in this analysis [26]. Additionally, Yuan-b et al. (2025b) analyzed European-ancestry GWAS data and found that each 1-SD increase in BMI (OR = 1.94; 95% CI: 1.23–3.08), waist circumference (OR = 2.40; 95% CI: 1.36–4.24), fasting glucose (OR = 5.01; 95% CI: 2.00–12.55), and two-hour glucose (OR = 3.17; 95% CI: 1.63–6.18) significantly elevated RVO risk, whereas higher whole-body fat-free mass was protective (OR = 0.45; 95% CI: 0.26–0.80) [28]. Multivariable MR that adjusted for mutual confounding and five cardiovascular covariates confirmed independent, directionally consistent effects for BMI (OR = 2.21; 95% CI: 1.39–3.52), fasting glucose (OR = 5.49; 95% CI: 1.99–15.14), and two-hour glucose (OR = 2.20; 95% CI: 1.23–3.95), while reinforcing the protective role of fat-free mass (OR = 0.39; 95% CI: 0.16–0.94). Huang et al. performed a two-sample MR analysis to evaluate the causal impact of genetically predicted T2DM on RVO risk and found a strong positive association: the primary IVW estimate showed an OR = 2.82 (95% CI: 2.07–3.85), which remained significant after multivariable adjustment (adjusted OR = 1.75; 95% CI: 1.24–2.47) [19].

The evidence for BMI and obesity traits is further complemented by the findings of Zheng et al., who reported significant associations between genetically predicted higher LDL-C (OR = 1.23, 95% CI: 1.05–1.44) and total cholesterol (OR = 1.44, 95% CI: 1.08–1.92) with increased RVO risk, while higher HDL-C was associated with a protective effect (OR = 0.81, 95% CI: 0.66–0.99) [18]. No significant effect was observed for triglycerides (OR = 1.10, 95% CI: 0.88–1.38).

### Other risk factors

Evidence for other pathways was mixed. Fan et al. conducted a comprehensive bidirectional MR analysis to investigate the causal effects of genetically predicted [25(OH)D] levels on multiple ocular disorders, including RVO [20]. The analyses revealed no statistically significant causal association between vitamin D levels and RVO risk (IVW OR = 1.34, 95% CI: 0.80–2.23 in discovery; OR = 1.27, 95% CI: 0.75–2.14 in validation).

Yuan-a et al. (2025a) applied a rigorous two-sample MR framework to 11 genetically proxied coagulation and platelet traits [27]. The study found that higher genetically predicted levels of coagulation factor III (OR = 0.58, 95% CI: 0.35–0.96) and factor VII (OR = 0.74,95% CI: 0.57–0.97) were causally linked to a 26–42% reduction in RVO odds, while elevated factor VIII increased risk (OR = 1.31, 95% CI: 1.07–1.60). Among platelet indices, greater mean platelet volume (OR = 0.78, 95% CI: 0.62–0.99) and platelet crit (OR = 0.74, 95% CI: 0.56–0.98) were protective. No robust associations emerged for FV, FX, FXI, plasmin, platelet count, or PDW. Sensitivity analyses (weighted-median, MR-Egger) produced concordant effect directions and showed no evidence of heterogeneity or directional pleiotropy, reinforcing a specific etiological role for extrinsic pathway factors and platelet morphology in RVO development.

In an MR study, Lei et al. found that six gut microbiota classes showed consistent, directionally positive causal effects on RVO risk. These included class Bacilli (discovery OR = 1.34, 95% CI: 1.12–1.59; validation OR = 1.17, 95% CI: 1.04–1.32), Lactobacillales (discovery OR = 1.31, 95% CI: 1.09–1.59; validation OR = 1.17, 95% CI: 1.03–1.32), Streptococcaceae (discovery OR = 1.46, 95% CI: 1.08–1.99; validation OR = 1.16, 95% CI: 1.03–1.32), genus *Clostridium innocuum* group (discovery OR = 1.17, 95% CI: 1.02–1.34; validation OR = 1.09, 95% CI: 1.00–1.19), genus Family XIII AD3011 group (discovery OR = 5.10, 95% CI: 2.05–12.65; validation OR = 1.34, 95% CI: 1.09–1.65), and genus *Subdoligranulum* (discovery OR = 1.31, 95% CI: 1.06–1.61; validation OR = 1.86, 95% CI: 1.27–2.74) [25]. Multiple-method sensitivity analyses (weighted median/mode) reproduced these estimates, and Bacilli plus Family XIII AD3011 withstood FDR correction, underscoring their robustness.

Among the included studies, only Yuan-b et al. (2025b) conducted multivariable MR analyses. The majority of other studies employed standard univariable MR approaches such as IVW, MR-Egger, and weighted median methods.

### Robustness of associations

Ocular traits (IOP and glaucoma) were supported by robust evidence, with consistent, statistically significant results across all MR methods and no indication of pleiotropy or heterogeneity. Blood pressure and hypertension showed a mix of robust and probable evidence, with strong IVW estimates and supporting sensitivity analyses in most cases. However, one IVW estimate for TG lacked significance, resulting in an “insufficient” rating. Lipid traits (HDL, LDL, TC, TG) demonstrated heterogeneity in direction and strength of effects, HDL-C consistently showed protection (robust), while LDL-C and TC were probable, and TG showed suggestive or null effects. Glycemic traits (fasting glucose, 2-hour glucose, HbA1c) also varied: robust evidence was found for acute hyperglycemia (FG, 2 h-G), while HbA1c showed conflicting directions (suggestive), and others showed no consistent effect. T2DM had one robust, one probable, and one suggestive result, reflecting consistent directionality but varied sensitivity support, where the association in Jiang et al. dropped to null after adjusting for multiple testing [24]. Anthropometric traits such as BMI and waist circumference showed robust and probable associations with RVO, while lean mass had protective effects. Coagulation and platelet traits yielded robust findings for factors III and VII and platelet indices, while others, like FXI or plasmin, showed no association. Vitamin D showed no significant effect across any method, with one analysis lacking full sensitivity testing, resulting in an “insufficient” and “non-evaluable” classification. Finally, gut microbiota taxa such as Bacilli and Family XIII AD3011 showed robust and consistent effects, while others showed weaker or partial evidence, and one analysis lacked sensitivity testing, resulting in a non-evaluable grading (Table 3).

**Table 3:** Robustness of associations between different exposures and the risk of RVO and its subtypes

Exposure category	Robust evidence	Probable evidence	Suggestive evidence	Insufficient evidence	Non-evaluable
Ocular traits (IOP, Glaucoma)	2 (100.0%)	0 (0.0%)	0 (0.0%)	0 (0.0%)	0 (0.0%)
Blood pressure & Hypertension	2 (40.0%)	2 (40.0%)	0 (0.0%)	1 (20.0%)	0 (0.0%)
Lipids (HDL, LDL, TC, TG)	1 (20.0%)	2 (40.0%)	1 (20.0%)	1 (20.0%)	0 (0.0%)
Glycemic traits (FG, 2 h-glucose, HbA1c)	2 (28.6%)	2 (28.6%)	1 (14.3%)	2 (28.6%)	0 (0.0%)
Type 2 Diabetes	1 (33.3%)	1 (33.3%)	1 (33.3%)	0 (0.0%)	0 (0.0%)
Anthropometric (BMI, WC, Lean Mass)	2 (33.3%)	2 (33.3%)	0 (0.0%)	2 (33.3%)	0 (0.0%)
Coagulation & Platelet traits	2 (33.3%)	2 (33.3%)	1 (16.7%)	1 (16.7%)	0 (0.0%)
Vitamin D	0 (0.0%)	0 (0.0%)	0 (0.0%)	2 (66.7%)	1 (33.3%)
Gut Microbiota	2 (40.0%)	1 (20.0%)	1 (20.0%)	0 (0.0%)	1 (20.0%)

IOP = Intraocular Pressure; HDL = High-Density Lipoprotein; LDL = Low-Density Lipoprotein; TC = Total Cholesterol; TG = Triglycerides; FG = Fasting Glucose; HbA1c = Hemoglobin A1c; T2DM = Type 2 Diabetes Mellitus; BMI = Body Mass Index; WC = Waist Circumference; RVO = Retinal Vein Occlusion

## Discussion

Our systematic review of twelve MR studies reveals a convergent risk profile for RVO that centers on ocular pressure, systemic hemodynamics, and metabolic dysregulation. Genetically elevated IOP and lifelong glaucoma liability were consistently associated with higher odds of RVO, although the available MR studies did not specifically test for non-linear dose–response relationships. Hypertension emerged as an independent systemic driver, while metabolic domains such as adiposity (BMI and waist circumference) and acute glycemic traits (fasting and two-hour glucose) robustly increased RVO risk. In contrast, greater lean mass exerted a protective influence. Findings for HbA1c were inconsistent. While diabetes overall was associated with increased RVO risk, some analyses suggested that higher genetically proxied HbA1c levels might be linked to reduced risk. However, these results should be interpreted with caution due to potential pleiotropy and wide confidence intervals. Lipids, particularly higher LDL-C and total cholesterol, displayed moderate harmful effects, whereas HDL-C appeared protective. Beyond these core pathways, limited but intriguing evidence implicated coagulation factors (protective roles for factors III & VII, harmful for VIII) and selected gut-microbial taxa, while vitamin D showed no causal link. Collectively, these findings underscore the multifactorial vascular and metabolic etiology of RVO and point to blood pressure control, weight management, and glycemic regulation as key preventive targets.

Most of the associations found in our review align with findings from observational studies. Importantly, the relationship between RVO and glaucoma may be bidirectional: glaucoma increases susceptibility to RVO, while RVO can lead to neovascular glaucoma. The association between glaucoma and RVO is well documented with a recent meta-analysis of observational studies finding the odds ratio of RVO in glaucoma patients to be 4.01 (95% CI: 3.28–4.91) [30]. Chang et al. found in a retrospective cohort elevated risk of RVO in patients with hypertension (adjusted HR = 1.50, 95% CI = 1.36–1.65) and diabetes (adjusted HR = 1.76, 95% CI = 1.61–1.93), even after adjusting for possible covariates [31], which is consistent with our findings on blood pressure and T2DM. Recent evidence also suggests that the use of glucagon-like peptide-1 receptor agonists (GLP-1RAs) in T2DM patients may confer a protective effect against RVO compared to dipeptidyl peptidase-4 (DPP-4) inhibitors [32]. The evidence on BMI and obesity also aligns with the broader literature, although findings remain controversial. Paik et al. reported a differential effect of BMI on the incidence of RVO based on diabetes status, with lower BMI being protective in patients without diabetes but harmful in those with diabetes [33]. Notably, the only significant MR evidence on BMI in our study was derived mainly from Yuan-b et al.‘s study, whose multivariate analysis did not adjust for diabetes [28]. In contrast, Jiang et al. reported a non-significant inverse association (OR = 0.845, 95% CI = 0.622–1.148), further supporting the presence of a complex association [24, 28].

The evidence on coagulation factors was found to be robust in our study, aligning with previous findings on the association between coagulation disorders and risk of RVO [34]. In a small case-control study of 54 patients with RVO and 54 age- and sex-matched controls, Sahu et al. reported a strong association between vitamin D deficiency and RVO, with an OR of 10.558 (CI = 2.34–47.50) [35]. Similarly, a meta-analysis by Daneshevar et al., which included 589 participants, concluded that vitamin D deficiency increased the risk of RVO (OR = 14.51; 95% CI: [1.71, 122.59], *P* = 0.014) [36]. However, despite this growing observational evidence, the included studies reported no causal association between vitamin D levels and RVO in MR studies. It is important to note that much of the observational evidence is derived from small studies with wide confidence intervals, limiting the overall strength of evidence. The evidence on gut microbiota adds new insights to an emerging trend in exploring microbial influences in ophthalmic and neurologic conditions. Ai et al. reported that the RVO group had higher levels of *Escherichia-Shigella* (*P* < 0.05) and lower abundance of *Parabacteroides* (*P* < 0.01) compared with the non-RVO group [37, 38].

Although all three MR studies (Jiang et al., Nie et al., and Yuan-b et al. [2025b]) examined the genetic association between glycemic traits and RVO, they reached different conclusions [24, 26, 28]. The differences among the three MR studies largely stem from variations in genetic instrument strength, exposure definitions, outcome datasets, and statistical approaches. Yuan-a et al. (2025a) leveraged large-scale GWAS data with strong SNP instruments (e.g., 423 SNPs for BMI, 27 for fasting glucose) and applied false discovery rate correction [27]. This approach allowed them to detect significant causal effects of overall adiposity and acute glycemic traits on RVO risk, with BMI (OR = 1.94, 95% CI 1.23–3.08) and fasting glucose (OR = 5.01, 95% CI 2.00-12.55) showing strong associations, but not HbA1c. Their multivariable MR analysis confirmed BMI, whole-body fat-free mass, fasting glucose, and two-hour glucose as independent risk factors for RVO. Nie et al. conducted only univariate analysis using a wider phenome-wide approach, with smaller SNP sets and less specific RVO outcome definitions, finding a modest protective effect of HbA1c but null effects for BMI and fasting glucose [26]. Jiang et al. examined a broader range of cardiometabolic and ocular traits, including all “retinal vascular occlusion” codes, but applied stricter Bonferroni corrections and adjusted for hypertension and glaucoma [24]. This resulted in hypertension and glaucoma remaining significant, while BMI and glycemic traits lost significance, which may be due to the adjustment. It is worth noting that Yuan-b et al. (2025b) used a more relaxed threshold (*p* < 5 × 10 − 6) to screen for SNPs compared to the (*P* < 5 × 10 − 8) threshold used in Nie et al. and Jiang et al. [24, 26, 28]. These methodological and analytical differences explain the divergent findings and highlight the need for harmonized instruments, exposure granularity, and outcome definitions in future MR studies on RVO.

Mechanistically, these signals align with Virchow’s triad of (i) venous stasis -elevated intraocular pressure (IOP) can mechanically impede retinal venous outflow at the lamina cribrosa and within the confined optic nerve head, slowing flow and predisposing to clot [39]; (ii) endothelial injury and structural narrowing-systemic hypertension and hyperlipidemia accelerates venous compression, turbulent shear, and microendothelial damage that favor thrombosis [40]; and (iii) hypercoagulability and microvascular fragility-glucose dysregulation (hyperglycemia/insulin resistance) promotes basement-membrane thickening, oxidative stress, low-grade inflammation, and a prothrombotic milieu (e.g., platelet activation/fibrinogen), [41, 42]. These processes likely interact, and previous reviews have further supported these mechanisms and findings [5, 43].

Our review offers a rigorous and transparent synthesis of MR evidence for RVO. Dual-reviewer screening, data extraction, and STROBE-MR-based quality appraisal minimized selection and abstraction bias, while the uniformly high checklist scores (> 85% for all studies) attest to the quality of reporting in the included literature. By applying grading rules that integrate effect direction, statistical significance, and concordance across multiple sensitivity estimators, we provide a nuanced hierarchy of causal credibility of the evidence. However, this review was limited by the few included studies and databases, preventing us from conducting a meta-analysis. All included studies were restricted to individuals of European ancestry, limiting generalizability to other populations and precluding ethnicity-specific insights. Several exposures (e.g., HbA1c, gut microbiota) were instrumented with smaller SNP sets or liberal significance thresholds, raising concerns about weak instrument bias and residual pleiotropy. Reliance on registry-based diagnostic codes may introduce bias if diagnoses are influenced by comorbid glaucoma or diabetes that are genetically linked to exposures, and this shall be taken into consideration when interpreting our results. Environmental modifiers such as smoking, medication use, and systemic inflammation may interact with genetic predisposition to influence RVO risk, but due to the paucity of data, this aspect should be explored in future research. MR estimates reflect lifelong genetic predisposition; temporality and age-related penetrance were not captured. Exposures such as elevated intraocular pressure or acute glycemic spikes may have age-dependent effects. All included studies pooled CRVO and BRVO into a single RVO outcome, without conducting subtype-specific analyses.

Genetically supported associations that we found suggest potential targets for risk assessment and preventive focus, but further evidence is needed before translation to practice. In real-world settings, our findings support screening prioritization for patients with diabetes, obesity, hyperlipidemia, and increased IOP for RVO. Additionally, therapeutic strategies should focus on controlling these risk factors. Future work should prioritize multi-ancestry GWAS to generate stronger, ancestry-specific instruments and validate causal pathways in non-European populations. Harmonized phenotyping, distinguishing CRVO from BRVO, exploring their specific risk factors, and incorporating retinal imaging biomarkers would refine outcome definitions and strengthen causal inference. Multivariable MR frameworks that jointly model blood pressure, adiposity, lipids, and glycemic traits are needed to clarify the contributions of correlated cardiometabolic pathways. Additionally, for ophthalmic traits, due to the paucity of data, future studies should explore the specific roles of traits such as retinal vessel caliber, optic nerve morphology, and choroidal thickness in RVO risk. Risk may escalate beyond thresholds for traits like blood pressure, BMI, and glucose, and our findings shall be correlated with future studies examining the dose-response relationships of these factors. Additionally, exploring whether sex would affect these associations could be of interest. Finally, integrating molecular-quantitative trait locus data (e.g., proteomics or metabolomics) and longitudinal electronic health record cohorts may identify druggable targets and provide translational traction for preventive interventions.

## Conclusion

Synthesizing evidence from twelve MR studies, the included studies reported that elevated IOP, glaucoma liability, hypertension, adiposity, and acute hyperglycemia are convincing causal drivers of RVO, whereas higher lean mass and favorable lipid or coagulation profiles confer protection. These findings support a multifactorial vascular–metabolic model of RVO etiology and underscore the potential of blood pressure control, weight management, and glycemic regulation in risk reduction. As larger, more diverse genetic datasets emerge, refined MR approaches will further illuminate mechanisms and inform targeted prevention strategies for this sight-threatening condition.

## Supplementary Information

Below is the link to the electronic supplementary material.

Supplementary Material 1

## Data Availability

The data used in this review were extracted from published sources. Extracted datasets used during the current study are available from the corresponding author upon reasonable request.

## Abbreviations

RVO: Retinal vein occlusion
MR: Mendelian randomization
IOP: Intraocular pressure
POAG: Primary open-angle glaucoma
CRVO: Central retinal vein occlusion
BRVO: Branch retinal vein occlusion
T2DM: Type 2 diabetes mellitus
GWAS: Genome-wide association studies
HDL-C: High-density lipoprotein cholesterol
LDL-C: Low-density lipoprotein cholesterol
TG: Triglycerides
TC: Total cholesterol
25(OH)D: Serum 25-hydroxyvitamin D
IS: Ischemic stroke
MI: Myocardial infarction
BMI: Body mass index
IVW: Inverse variance weighted
SD: Standard deviation
CVDs: Cardiovascular diseases
GLP-1RAs: Glucagon-like peptide-1 receptor agonists
DPP-4: Dipeptidyl peptidase-4 b
