# Supplementary material for: Unraveling causal pathways in retinal vein occlusion: a systematic review of Mendelian randomization studies
Source: Int J Retina Vitreous. 2025 Dec 3;11:133. doi: 10.1186/s40942-025-00753-7 (PMC12676853; doi:10.1186/s40942-025-00753-7)
Supplement: Supplementary file 1 — Supplementary Material 1 [file 40942_2025_753_MOESM1_ESM.docx]

Supplementary Material

Unraveling Causal Pathways in Retinal Vein Occlusion: A Systematic Review of Mendelian Randomization Studies

**Supplementary Table 1**: Search strategy for the included studies

| Database | Search strategy |
| --- | --- |
| PubMed | ("retinal vein occlusion" OR "RVO" OR "central retinal vein occlusion" OR "branch retinal vein occlusion") AND ("Mendelian randomization" OR "Mendelian randomisation" OR "genetic instrumental variable" OR "MR study" OR "two-sample MR" OR "one-sample MR" OR "genetic proxy"). |
| Scopus | ( ALL ( "Mendelian randomization" OR "Mendelian randomisation" OR "genetic instrumental variable" OR "MR study" ) ) AND ALL ( ( "retinal vein occlusion" OR "RVO" OR "central retinal vein occlusion" OR "branch retinal vein occlusion" ) ) |
| Web of Science | ( "Mendelian randomization" OR "Mendelian randomisation" OR "genetic instrumental variable" OR "MR study" ) (All Fields) and ( "retinal vein occlusion" OR "RVO" OR "central retinal vein occlusion" OR "branch retinal vein occlusion" ) (All Fields) |
| Embase | ("retinal vein occlusion" or "RVO" or "central retinal vein occlusion" or "branch retinal vein occlusion") and ("Mendelian randomization" or "Mendelian randomisation" or "genetic instrumental variable" or "MR study" or "two-sample MR" or "one-sample MR" or "genetic proxy")).mp. [mp=title, abstract, heading word, drug trade name, original title, device manufacturer, drug manufacturer, device trade name, keyword heading word, floating subheading word, candidate term word] |
